# Interactions between assembly order and temperature can alter both short- and long-term community composition

**DOI:** 10.1002/ece3.901

**Published:** 2013-11-28

**Authors:** Christopher F Clements, Philip H Warren, Ben Collen, Tim Blackburn, Nicholas Worsfold, Owen Petchey

**Affiliations:** 1 Department of Animal and Plant Sciences, University of SheffieldSheffield, S10 2TN, U.K; 2 Centre for Biodiversity & Environment Research, University College LondonGower Street, London, WC1E 6BT, U.K; 3 Institute of Zoology, ZSLRegent's Park, London, NW1 4RY, U.K; 4 Distinguished Scientist Fellowship Program, King Saud UniversityP.O. Box 2455, Riyadh, 1145, Saudi Arabia; 5 Department of Life Sciences, University of BedfordshireLuton, LU1 3JU, U.K; 6 Institute of Evolutionary Biology and Environmental Studies, The University of ZurichZurich, CH-8057, Switzerland

**Keywords:** Community assembly, global warming, priority effects, protist microcosm, temperature.

## Abstract

Both the order in which species arrive in a community, and environmental conditions, such as temperature, are known to affect community structure. Little is known, however, about the potential for, and occurrence of, interactions between assembly history and the environment. Of particular, interest may be the interaction between temperature and community assembly dynamics, especially in the light of predicted global climatic change and the fundamental processes that are governed, through metabolic rate, by an individual's environmental temperature. We present, to our knowledge, the first experimental exploration of how the influence of assembly history, temperature, and the interaction between the two alters the structure of communities of competitors, using small-scale protist microcosm communities where temperature and assembly order were manipulated factorially. In our experiment, the most important driver of long-term abundance was temperature but long-lasting assembly order effects influenced the relationship between temperature and abundance. Any advantage of early colonization proved to be short-lived, and there was rarely any long-term advantage to colonizing a habitat before other species. The results presented here suggest that environmental conditions shape community composition, but that occasionally temperature could interact with the stochastic nature of community assembly to significantly alter future community composition, especially where temperature change has been large. This could have important implications for the dynamics of both rare and invasive species.

## Introduction

Species' abundances and distributions are predicted to change substantially under anthropogenically driven climate change (Condit et al. 1996; Iverson and Prasad 1998; Perry et al. 2005). Current predictions suggest that global temperatures are set to rise between 1.1 and 6.4°C over the next 100 years (IPCC 2007), with potentially profound impacts on ecosystems and communities worldwide (Kasischke et al. 1995; Thomas et al. 2004; Pandolfi et al. 2011). Temperature can directly determine which species survive in a habitat (Ferguson 1958; Southward 1958), but can also alter individual, population, and community scale processes, which in turn can have complex cascading effects (Kratina et al. 2012). For example, as the metabolic requirements of an organism increase with increasing temperature, resource competition will intensify, so higher temperatures may result in greater interspecific interaction strengths (Gresens et al. 1982; Sanford 1999, 2002; Englund et al. 2011). Jiang and Morin (2004) showed that a temperature difference of just 2°C reversed competition between two ciliate protozoa; initial rapid competitive exclusion was replaced by co-existence. This change in community structure could in turn impact food web stability (Rall et al. 2009) and may have the potential to alter ecosystem function. For example, a shift in community composition caused by temperature change has been shown to alter cyanobacterial diversity, with, in some instances, a shift to toxin-producing species (Kleinteich et al. 2012).

Another way in which community composition can be altered is through assembly order effects, where the order in which species colonize a habitat can influence the competitive ability or abundance of a species (Shorrocks and Bingley 1994; Almany 2003; Louette and De Meester 2007; Chase 2010). Such effects have been demonstrated in model (Atkinson and Shorrocks 1981; Law and Morton 1996), small-scale experimental (Drake 1991; Fukami and Morin 2003; Warren et al. 2003), and field systems (Weslien et al. 2011; Dickie et al. 2012). Such assembly order effects can be profound: arriving at a patch even marginally before another may transform an inferior competitor into a superior one (Shorrocks and Bingley 1994), allowing a species to persist where it might otherwise be excluded. Moreover, because species may be competitively excluded based on the order in which they arrive in a habitat, assembly order could also play an important role in the survival of species at a local or regional scale (Shorrocks and Bingley 1994; Chase 2010).

Recent work has started to look at how environmental factors, including disturbance (Jiang and Patel 2008) and productivity (Chase 2010), may alter the role of community assembly processes. It has been suggested that assembly order effects are most likely to be important when the species pool is large and the habitat is both productive and stable (Chase 2003). So far, however, despite the acknowledged importance of temperature effects on biological processes and the importance of understanding the consequences of environmental warming, the interaction between assembly order and temperature has received little attention.

In this study, we investigate the interaction between temperature and assembly order using a laboratory experiment where temperature and the assembly order of a three-species protist community were manipulated factorially to assess: (i) how temperature alters the advantage of initially colonizing a habitat, (ii) whether colonizing a habitat early has a long-term advantage for a species, (iii) whether the order in which species invade a habitat can modify the strength, and direction, of the effect of temperature on species abundance.

## Methods

We performed a two-way factorial manipulation of assembly order and temperature in microcosm communities assembled with three species of bactiverous ciliate protozoa: *Blepharisma japonicum*, *Paramecium caudatum*, and *Loxocephalus sp*. (subsequently denoted by the letters B, P, and L). These three species were chosen because they compete for similar resources, because they can co-occur in natural environments, and they are morphologically very distinct, facilitating accurate sampling. One species, *Blepharisma japonicum*, is known to be able to form enlarged predatory morphs; however, over the course of the experiment, none of these morphs were observed, and previous experiments have shown that predatory morphs form most frequently when nutrients are low (half the concentration used in this experiment) and populations persist for an elongated period of time (Clements, pers. obs.). Therefore, we feel justified in considering the communities presented here as communities of competitors only.

Microcosms consisted of petri dishes (diameter 100 mm, height 20 mm) containing 50 mL of medium, composed of Chalkley's solution (Thompson et al. 1988) and 0.2 g/L crushed protist pellets (Carolina Biological Supply, Burlington, NC) autoclaved together. Medium was batch inoculated with the bacteria *Serratia marcescens* and *Bacillus cereus*, and incubated for 7 days at 18.5°C to allow bacterial populations to develop. Medium was then mixed and split among the microcosms (experimental day 0) when a single wheat seed was added to each to provide an additional source of nutrients.

Protists were added sequentially at 7-day intervals (on days 0, 7, and 14). On each day, a sample of high-density stock culture containing ∼30 individuals of each species was added to each microcosm. Assembly orders covered all seven possible combinations of species invasions: BPL (i.e., B on day 0, then P on day 7, then L on day 14), BLP, PBL, PLB, LBP, LPB, and, a control group, ALL, where all three species were added at day 0. Each assembly order was replicated three times at each of six temperatures (11, 14, 17, 20, 23, and 26°C) in 6 individual incubators, giving a total of 126 microcosms. The microcosms were randomly assigned a position on a shelf within each incubator. As the incubator facility was shared, with other experiments being run concurrently with this one, we were unable to switch treatments between incubators during the experiment to guard against possible incubator effects, although we have no reason to suspect such effects were likely to be present. The abundances of all species present in each microcosm were sampled on days 7, 14, 21, 42, and 70. A setup error in all three replicates of BPL at 23°C meant that this treatment had to be excluded.

Sampling to estimate species abundances was based on Lawler and Morin (1993). Microcosms were mixed thoroughly, and then known volumes (between 0.2 and 0.5 mL) were sampled using a Gilson pipette. Individuals of each species present in these subsamples were counted under a stereoscopic microscope. If no individuals of a species were observed, the microcosm was resampled up to three times. For rare species, the entire microcosm was placed under the microscope and searched, with a species being recorded as extinct if no individuals were observed after 5 min of searching. All sampled medium was returned to the microcosm. Evaporative loss was checked on a weekly basis, and microcosms were topped up to 50 mL with distilled water as required. No additional nutrients were added to the microcosms, and no replacement of medium (save for evaporative loss) occurred.

Count data recorded during the experiment were highly skewed, with some species (especially *Loxocephalus*) having high numbers of extinctions (i.e., zero densities) whilst also having some populations at extremely high densities (>11,000 in a microcosm). Consequently, generalized linear models (GLM), with Gaussian or quasi-Poisson distribution families, were used to model abundances of *Blepharisma* and *Paramecium*. A GLM with zero-inflated negative binomial distribution family (henceforth ZNBR) was used to model the abundance of *Loxocephalus* due to the high proportion of zero counts and overdispersion of the observed data (Ridout et al. 2001). Analyses were repeated for data from days 42 and 70, the last 2 days at which microcosms were sampled for abundance data. This allowed us to investigate long-term community structure and how the relative strength of factors influencing species abundance changed over time.

We calculated the strength of any advantage of colonizing a habitat 1st, 2nd, or 3rd as the difference in abundance between treatments where the species were added sequentially and the mean abundance in the control treatment where all the species were added simultaneously (i.e., with no assembly order effects). This gave six differences (one from each of the three replicates of the two treatments where a species was added 1st, 2nd, or 3rd); we then calculated the mean and standard error of these.

All statistical analyses were carried out using R (R Core Team 2013).

## Results

### Analysis of abundance patterns at days 42 and 70

**Figure 1:**
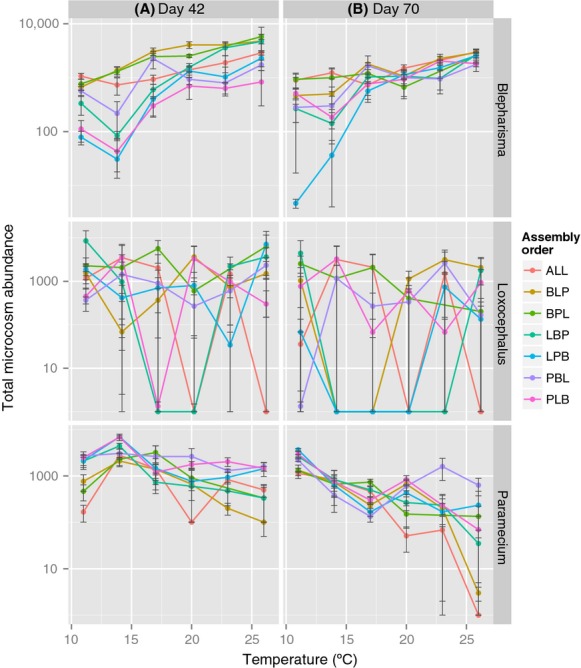
Mean abundances of the three species at day 42 (A) and day 70 (B) for each assembly order and temperature treatment, highlighting the individual species responses to temperature. Line color indicates assembly order. Bars represent ±1 standard error.

Abundances of *Paramecium* were significantly negatively, and *Blepharisma* significantly positively, correlated with temperature (Fig. 1; Tables 1, 2). This general pattern held for both day 42 and day 70, although the strength of the effect of temperature on species abundance tended to be higher at day 70 than at day 42 (Fig. 1; Tables 1, 2).

**Table 1:** Analysis of deviance of generalized linear models fitted to the abundance of *Paramecium* at days 42 and 70. Statistically significant interaction coefficients of generalized linear models presented as Temp∼ the relevant assembly order

	Day 42				Day 70			
		
Term	Error	df	*F*-value	*P*-value	Error	df	*F*-value	*P*-value
Temp	**G**	**1, 120**	**24.95**	**<0.001**	**q-P**	**1, 120**	**138.22**	**<0.001**
Ass. Or.	**G**	**6, 114**	**5.41**	**<0.001**	**q-P**	**6, 114**	**2.42**	**<0.05**
Interaction	G	6, 108	1.71	>0.05	**q-P**	**6, 108**	**3.45**	**<0.01**

	Error	Estimate	*t*-value	*P*-value	Error	Estimate	*t*-value	*P*-value
Temp∼BPL	**G**	**−0.17**	**2.12**	**<0.05**	q-P	0.08	0.94	>0.05
Temp∼LBP	**G**	**−0.17**	**2.30**	**<0.05**	q-P	−0.01	0.08	>0.05
Temp∼PBL	G	−0.04	0.50	>0.05	**q-P**	**0.19**	**2.58**	**<0.05**

df, degrees of freedom; Error structures are: “G”, Gaussian; q-P, quasi-Poisson.

95% significance is highlighted in bold.

**Table 2:** Analysis of deviance of generalized linear models fitted to the abundance of *Blepharisma* at days 42 and 70. Statistically significant interaction coefficients of generalized linear models presented as Temp∼ the relevant assembly order

	Day 42				Day 70			
		
Interaction (Temp∼)	Error	df	F-value	P-value	Error	df	F-value	P-value
Temp	**q-P**	**1, 120**	**149.06**	**<0.001**	**G**	**1, 120**	**152.10**	**<0.001**
Ass. Or.	**q-P**	**6, 114**	**21.70**	**<0.001**	**G**	**6, 114**	**4.87**	**<0.001**
Interaction	**q-P**	**6, 108**	**3.44**	**<0.01**	**G**	**6, 108**	**2.36**	**<0.05**

	Error	Estimate	*t*-value	*P*-value	Error	Estimate	*t*-value	*P*-value
Temp∼LBP	**q-P**	**0.13**	**3.08**	**<0.01**	G	0.64	1.03	>0.05
Temp∼LPB	**q-P**	**0.11**	**2.16**	**<0.05**	**G**	**1.68**	**2.71**	**<0.01**

df, degrees of freedom; Error structures are: “G”, Gaussian; q-P, quasi-Poisson.

95% significance is highlighted in bold.

Assembly order also altered the abundances of both *Paramecium* and *Blepharisma* (Fig. 1; Tables 1, 2), although the strength of this effect decreased from day 42 to day 70, and accounted for less of the variation in abundance than the effect of temperature (Tables 1, 2). *Blepharisma* at day 42 showed particularly clear differences in abundance between assembly orders (Fig. 1A), with some assembly orders having consistently lower or higher abundances than others (e.g., PLB, BLP, Fig. 1A).

In addition to directly altering *Paramecium* and *Blepharisma* population sizes, assembly order could also alter the relationship between a species' abundance and temperature (Fig. 1; Tables 1, 2). This interaction could either increase or decrease the strength of the effect of temperature (Fig. 1), and in some cases, this interaction accounted for a two order of magnitude difference in the abundance of *Blepharisma* (e.g., the assembly order LPB at day 70, Fig. 1B) and *Paramecium* (e.g., the assembly order PBL at day 70, Fig. 1B). These interactive effects are particularly clear, but less frequent, at day 70 (Fig. 1B).

**Figure 2:**
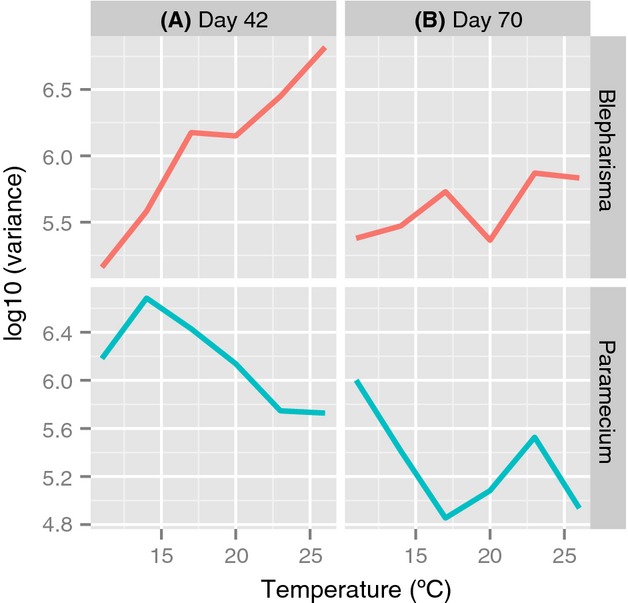
Variation (between assembly orders) in the total abundance of each species in the microcosms, as a function of temperature at day 42 (A) and day 70 (B).

Variation in abundance among the different assembly orders correlated with temperature, with the direction and strength of this correlation dependant on both species identity and the time since community assembly (Fig. 2). *Blepharisma* showed a strong positive correlation between temperature and variance in abundances at day 42 (i.e., large differences between assembly orders, especially at higher temperatures) and a still positive, but weaker, relationship at day 70. *Paramecium* meanwhile showed exactly the opposite relationship, with temperature negatively correlating with variation in abundance between assembly orders, however, the strength of this relationship again decreased from day 42 to 70 (Fig. 2).

The abundances of *Loxocephalus* in each treatment exhibited little evidence of systematic trends at either day 42 or day 70, and abundances were not significantly affected by temperature, assembly order, or any interaction between the two. There was, however, a significant increase in zero counts (i.e., extinctions) with increasing temperature, and the assembly order BLP at day 70 (Table S1).

### Effect of sequential invasion on species abundances at days 42 and 70

**Figure 3:**
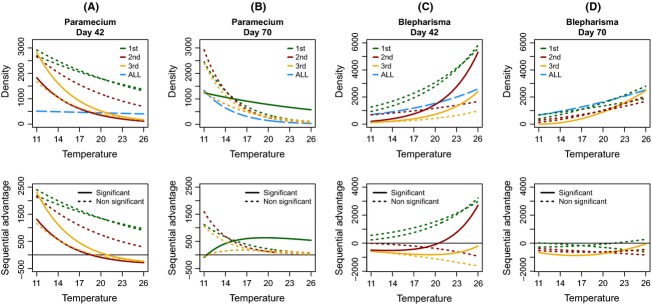
The abundances of *Blepharisma* and *Paramecium* were estimated at days 42 and 70, across the temperature range and assembly orders, from the coefficients of the fitted (generalized linear models) models (upper plots). The advantage of colonizing a habitat 1st, 2nd, or 3rd, as opposed to simultaneously with other species (i.e., the difference in abundance between sequential treatments and the treatment ALL) is plotted below. For each species, there are two assembly orders, and consequently two lines, where that species is added 1st, 2nd, or 3rd (e.g., *Loxocephallus* is added 1st in the assembly orders LBP and LPB, 2nd in the orders BLP and PLB, and 3rd in the orders BPL and PBL). “Significant” indicates a difference in abundance between an assembly order where species have been added sequentially, and the treatment ALL (where they have gone in simultaneously) that has a *P*-value <0.05. Assembly orders with significantly higher abundances all also exhibited significant interaction between temperature and assembly order (Tables S2, S3).

Whilst there appeared to be some advantage, in terms of increased abundance, of colonizing a habitat sequentially over colonizing simultaneously, there was not necessarily an advantage in colonizing earlier, and the magnitude of any advantage could also be modified by temperature (Fig. 3). Higher temperatures did not necessarily lead to a larger long-term advantage of colonizing a habitat early, rather species-specific responses to temperature often drove the magnitude and direction of assembly order effects at each temperature treatment (Fig. 3): *Blepharisma* was more abundant at higher temperatures, and *Paramecium* was less abundant.

Of the four assembly orders where *Blepharisma* and *Paramecium* were added before any other species (i.e., added 1st; BLP, BPL and PBL, PLB), the initial colonizers tended to have higher, but not significantly higher, abundances (Fig. 3). At day 70, only one assembly order showed significantly higher abundances of the initial colonizer: PBL (Fig. 1B, Table 1). *Loxocephalus* abundances appeared to be almost randomly distributed across temperatures and treatments, and so were excluded from the analysis of early colonization advantage at days 42 and 70.

Arriving at a habitat after initial colonization by another species (i.e., arriving 2nd or 3rd) could alter abundance (when compared to the treatment ALL), but the direction of this effect was species and temperature specific (Fig. 3). At day 42, *Paramecium* showed an advantage of being added to a microcosm 2nd or 3rd (Fig. 3A), but by day 70, these effects had disappeared (Fig. 3B, Table 1). For *Blepharisma*, however, there appeared to be some disadvantage of colonizing a habitat late (after the two other species); populations had significantly lower abundances at days 42 and 70 when added to a community 3rd, although this was to a large extent negated by higher temperatures (Fig. 3C and D, Table 2).

## Discussion

Although both temperature and assembly order are known to be important drivers of community composition (Shorrocks and Bingley 1994; Jiang and Morin 2004; Kleinteich et al. 2012), there has been little investigation of the potential interaction between these two factors. The experimental evidence presented here suggests that the effect of temperature on species abundances, and therefore community composition, can be contingent on the order of assembly of that community. This does not appear to be driven by an advantage of colonizing early, as we only occasionally found a significantly higher abundance of initial colonizers at days 42 and 70; however, those species that colonized later were often at a disadvantage. Furthermore, we showed that the strength of the interaction, and of the main effects of temperature and assembly sequence, is a function of both time and species identity. These findings have important implications for modeling the potential effects of future climate change on community structure and species distributions.

In line with previous findings, our experimental work shows that species-specific responses to temperature are a major determinant of abundance, and thus community composition (Figs. 1, 3). Over the period of this experiment (∼100 protist generations for these species at 20°C (Clements et al. 2013)), the strength of this temperature effect increased, possibly because there has been a greater period of time for inferior competitors to be excluded (Fig. 1, Tables 1, 2). In contrast, whilst there were assembly order effects (Tables 1, 2), the strength of these are species-specific and transient; the size of the assembly order effect decreased from day 42 to 70, a finding supported up by a decrease in the variance between assembly orders over the same period (Fig. 2). In addition, by day 70, the effect of assembly order was small when compared to the dominant effect of temperature (Tables 1, 2). Our results indicate that intermediate levels of environmental change may have the potential to mask assembly order effects, leading to multiple similar community types regardless of assembly history. However, greater levels of change may, occasionally, promote the prevalence of such effects as, within the 15°C temperature range of our experiment, assembly order effects were most evident where it was either hottest or coldest.

Whilst interactions between temperature and assembly order appear to be rare, where they do occur, they can significantly alter the long-term structure of a community (Fig. 1). Although the magnitude of this interaction effect is small when compared to the effect of temperature alone (Tables 1, 2), and whilst it is only present in two of the seven assembly orders at day 70, the impact on the abundance of a species can be dramatic (Fig. 1B, *Blepharisma* and *Paramecium*). Clearly, there is the potential for such significant increases or decreases in a species' abundance to have a substantial effect on a community, especially if the species affected is a key pollinator (Memmott et al. 2004) or an invasive alien (Lowe et al. 2000).

Accurately predicting the potential impacts of future climate change on global diversity requires knowledge of the effects temperature can have at a population, community, and ecosystem level (Cramer et al. 2001; Brown et al. 2004; Jiang and Morin 2004). Earlier work has identified the role of temperature and other abiotic factors in shaping a species' fundamental niche (Hutchinson 1957), and such fundamental niches provide the underpinnings for “climate envelope” approaches to estimating future species distributions in relation to climatic change (Davis et al. 1998). However, this approach has been criticized, as species exist within a realized niche that is defined not only by the abiotic conditions but also interactions between species (Davis et al. 1998), as well as stochastic processes such as dispersal (Mitikka et al. 2007). If the interactions between species, that shape the realized niche, are also altered by climatic change, then climate envelopes, and other models that fail to take into account temperature-dependent interspecific interactions, may provide misleading estimates of future species distributions or community composition (Davis et al. 1998). Such concerns seem well founded, as previously small shifts in temperature have been shown to interact with a species' specific thermal tolerance to reverse competition in model systems (Jiang and Morin 2004). Our results add to this body of knowledge by highlighting the interaction between a stochastic driver of community composition and environmental change, and the potential to dramatically under or over estimate a species' future abundance. However, further work is required to understand the mechanistic underpinnings of the interactions between temperature and assembly order presented here if we are to improve such predictive frameworks.

In conclusion, the results presented here suggest that our ability to understand how communities may react to climate change is complicated by species-specific responses to temperature, ephemeral effects of assembly order and, occasionally, complex interactions between the order in which species invade a habitat and their competitive ability, as well as the time frame over which this occurs. Incorporating such interactions, in addition to stochastic and deterministic drivers of community composition, in future modeling is essential if one aims to encompass the full range of potential climate driven future community states. Whilst this may sound daunting, some heart should be taken from the fact that long-term dynamics are generally driven by abiotic conditions, and the potential complexity added by strong priority effects, at least in this system, appears short-lived. Thus, understanding general patterns of diversity under climatic change may be feasible, but identifying when and where temperature and assembly order will interact to alter community composition is likely to remain challenging.

## Supporting Information

**Table S1.** Coefficients of zero-inflated negative binomial regression models fitted to the abundances of *Loxocephallus* at days 42 and 70.

**Table S2.** Full coefficients of generalized linear models fitted to the abundances of *Paramecium* at days 42 and 70.

**Table S3.** Full coefficients of generalized linear models fitted to the abundances of *Blepharisma* at days 42 and 70.
