# Supplementary material for: Interactions between assembly order and temperature can alter both short- and long-term community composition
Source: Ecol Evol. 2013 Nov 28;3(16):5201–8. doi: 10.1002/ece3.901 (PMC3892329; doi:10.1002/ece3.901)
Supplement: Supplementary file 1 [file ece30003-5201-SD1.pdf]

Supplementary information tables

Table S1. Coefficients of zero-inflated negative binomial regression models fitted to the abundances of Loxocephallus at days 42 and 70. Zero-inflated model is the effect of the factors on the number of zeros (i.e. extinctions) recorded in the data. 95% significance is highlighted in bold, “nb” refers to the negative binomial error structure.

	Day 42					Day 70				
	Error	Std. Er	Coef.	z-value	p-value	Error	Std. Er.	Coef.	z-value	p-value
Count model										
Temp	nb	0.13	0.03	0.23	>0.05	nb	0.14	0.03	0.24	>0.05
BLP	nb	2.49	-1.39	0.56	>0.05	nb	3.06	0.12	0.04	>0.05
BPL	nb	2.57	-0.52	0.20	>0.05	nb	2.92	2.29	0.79	>0.05
LBP	nb	2.45	1.20	0.49	>0.05	nb	2.91	1.40	0.48	>0.05
LPB	nb	2.44	-1.71	0.70	>0.05	nb	3.46	-3.46	1.00	>0.05
PBL	nb	2.58	-1.80	0.69	>0.05	nb	3.18	-1.16	0.37	>0.05
PLB	nb	2.87	0.47	0.16	>0.05	nb	2.88	1.70	0.59	>0.05
T~BLP	nb	0.15	0.03	0.22	>0.05	nb	0.17	-0.02	0.1	>0.05
T~BPL	nb	0.16	0.05	0.30	>0.05	nb	0.17	-0.16	0.93	>0.05
T~LBP	nb	0.15	-0.05	0.33	>0.05	nb	0.17	-0.05	0.29	>0.05
T~LPB	nb	0.15	0.08	0.53	>0.05	nb	0.19	0.09	0.50	>0.05
T~PBL	nb	0.16	0.05	0.35	>0.05	nb	0.18	0.02	0.14	>0.05
T~PLB	nb	0.17	-0.05	0.27	>0.05	nb	0.16	-0.11	0.67	>0.05
Zero-inflation model										
Temp	**nb**	**0.25**	**0.13**	**1.99**	**<0.05**	nb	0.12	0.10	1.14	>0.05
BLP	nb	-1.22	4.36	0.28	>0.05	nb	5.73	3.03	1.89	>0.05
BPL	nb	1.78	3.24	0.55	>0.05	nb	0.31	2.71	0.12	>0.05
LBP	nb	2.14	3.03	0.71	>0.05	nb	0.97	3.03	0.32	>0.05
LPB	nb	1.45	3.13	0.46	>0.05	nb	4.41	3.23	1.37	>0.05
PBL	nb	2.30	3.09	0.75	>0.05	nb	2.36	2.69	0.88	>0.05
PLB	nb	2.60	3.03	0.86	>0.05	nb	2.18	2.68	0.81	>0.05
T~BLP	nb	-0.06	0.21	0.28	>0.05	**nb**	**-0.32**	**0.16**	**2.03**	**<0.05**
T~BPL	nb	-0.17	0.17	0.97	>0.05	nb	-0.05	0.15	0.31	>0.05
T~LBP	nb	-0.15	0.16	0.90	>0.05	nb	0.00	0.17	0.01	>0.05
T~LPB	nb	-0.12	0.16	0.75	>0.05	nb	-0.19	0.17	1.15	>0.05
T~PBL	nb	-0.18	0.16	1.08	>0.05	nb	-0.14	0.14	0.94	>0.05
T~PLB	nb	-0.18	0.16	1.14	>0.05	nb	-0.12	0.14	0.84	>0.05

Table S2. Full coefficients of generalized linear models fitted to the abundances of *Paramecium* at days 42 and 70. 95% significance is highlighted in bold. Error structures are as follows: "G" = Gaussian, "q-P" = quasi-Poisson.

	Day 42					Day 70				
	Error	Coef.	Std. Er.	t-value	p-value	Error	Coef.	Std. Er.	t-value	p-value
Temp	G	-0.01	0.02	0.30	>0.05	**q-P**	**-0.24**	**0.07**	**3.66**	**<0.001**
BLP	G	1.20	0.63	1.90	>0.05	q-P	-0.95	1.20	0.79	>0.05
BPL	**G**	**1.36**	**0.64**	**2.11**	**<0.05**	q-P	-0.94	1.24	0.75	>0.05
LBP	**G**	**1.57**	**0.63**	**2.48**	**<0.05**	q-P	0.66	1.17	0.57	>0.05
LPB	G	1.07	0.63	1.69	>0.05	q-P	1.03	1.14	0.90	>0.05
PBL	G	0.86	0.63	1.36	>0.05	**q-P**	**-2.18**	**1.10**	**1.98**	**<0.05**
PLB	G	0.94	0.63	1.48	>0.05	q-P	0.25	1.12	0.22	>0.05
T~BLP	G	-0.06	0.63	1.89	>0.05	q-P	0.08	0.08	1.00	>0.05
T~BPL	**G**	**-0.07**	**0.03**	**2.12**	**<0.05**	q-P	0.08	0.09	0.94	>0.05
T~LBP	**G**	**-0.08**	**0.03**	**2.28**	**<0.05**	q-P	-0.01	0.08	0.08	>0.05
T~LPB	G	-0.03	0.03	0.97	>0.05	q-P	-0.02	0.08	0.27	>0.05
T~PBL	G	-0.01	0.03	0.38	>0.05	**q-P**	**0.19**	**0.07**	**2.58**	**<0.05**
T~PLB	G	-0.02	0.03	0.50	>0.05	q-P	0.03	0.08	0.41	>0.05

Table S3. Full coefficients of generalized linear models fitted to the abundances of *Blepharisma* at days 42 and 70. 95% significance is highlighted in bold. Error structures are as follows: “G” = Gaussian, “q-P” = quasi-Poisson.

	Day 42					Day 70				
	Error	Coef.	Std.Er.	t-value	p-value	Error	Coef.	Std.Er.	t-value	p-value
Temp	**q-P**	**0.09**	**0.03**	**3.22**	**<0.01**	**G**	**1.63**	**0.44**	**3.71**	**<0.001**
BLP	q-P	0.46	0.71	0.64	>0.05	G	-12.44	11.89	1.04	>0.05
BPL	q-P	-0.09	0.74	0.13	>0.05	G	5.57	12.12	0.46	>0.05
LBP	**q-P**	**-2.65**	**0.95**	**2.80**	**<0.01**	G	-21.18	11.89	1.78	>0.05
LPB	**q-P**	**-3.01**	**1.19**	**2.54**	**<0.05**	**G**	**-44.19**	**11.89**	**3.71**	**<0.001**
PBL	q-P	0.29	0.85	0.34	>0.05	G	-8.85	11.95	0.74	>0.05
PLB	q-P	-2.33	1.35	1.72	>0.05	G	-20.86	11.89	1.76	>0.05
T~BLP	q-P	0.01	0.03	0.36	>0.05	G	0.58	0.62	0.94	>0.05
T~BPL	q-P	0.03	0.03	1.00	>0.05	G	-0.47	0.65	0.73	>0.05
T~LBP	**q-P**	**0.13**	**0.4**	**3.08**	**<0.01**	G	0.64	0.62	1.03	>0.05
T~LPB	**q-P**	**0.11**	**0.05**	**2.16**	**<0.05**	**G**	**1.68**	**0.62**	**2.71**	**<0.01**
T~PBL	q-P	-0.03	0.04	0.69	>0.05	G	-0.01	0.63	0.02	>0.05
T~PLB	q-P	0.05	0.06	0.85	>0.05	G	0.61	0.62	0.99	>0.05
